# Molecular Basis of Differential B-Pentamer Stability of Shiga Toxins 1 and 2

**DOI:** 10.1371/journal.pone.0015153

**Published:** 2010-12-28

**Authors:** Deborah G. Conrady, Michael J. Flagler, David R. Friedmann, Bradley D. Vander Wielen, Rhett A. Kovall, Alison A. Weiss, Andrew B. Herr

**Affiliations:** Department of Molecular Genetics, Biochemistry and Microbiology, University of Cincinnati College of Medicine, Cincinnati, Ohio, United States of America; University of Queensland, Australia

## Abstract

*Escherichia* coli strain O157:H7 is a major cause of food poisoning that can result in severe diarrhea and, in some cases, renal failure. The pathogenesis of *E. coli* O157:H7 is in large part due to the production of Shiga toxin (Stx), an AB_5_ toxin that consists of a ribosomal RNA-cleaving A-subunit surrounded by a pentamer of receptor-binding B subunits. There are two major isoforms, Stx1 and Stx2, which differ dramatically in potency despite having 57% sequence identity. Animal studies and epidemiological studies show Stx2 is associated with more severe disease. Although the molecular basis of this difference is unknown, data suggest it is associated with the B-subunit. Mass spectrometry studies have suggested differential B-pentamer stability between Stx1 and Stx2. We have examined the relative stability of the B-pentamers in solution. Analytical ultracentrifugation using purified B-subunits demonstrates that Stx2B, the more deadly isoform, shows decreased pentamer stability compared to Stx1B (EC_50_ = 2.3 µM vs. EC_50_ = 0.043 µM for Stx1B). X-ray crystal structures of Stx1B and Stx2B identified a glutamine in Stx2 (versus leucine in Stx1) within the otherwise strongly hydrophobic interface between B-subunits. Interchanging these residues switches the stability phenotype of the B-pentamers of Stx1 and Stx2, as demonstrated by analytical ultracentrifugation and circular dichroism. These studies demonstrate a profound difference in stability of the B-pentamers in Stx1 and Stx2, illustrate the mechanistic basis for this differential stability, and provide novel reagents to test the basis for differential pathogenicity of these toxins.

## Introduction

The gastrointestinal pathogen *Escherichia coli* O157:H7 accounts for approximately 110,000 cases of disease, 3,200 hospitalizations and 60 deaths each year in the United States [1]. *E. coli* O157:H7 illness is most often characterized by self-limiting diarrhea; however, in approximately 5% of cases disease progresses to a severe, life-threatening sequela called hemolytic uremic syndrome (HUS) [2]. HUS is characterized by hemolytic anemia, thrombocytopenia, and renal failure [3]. Progression of disease to HUS occurs most often in pre-adolescent children and the elderly, and HUS is the most common cause of acute renal failure in children [3], [4]. Ingesting as few as 50 bacteria can lead to disease [5], and conventional antibiotic treatment has been reported to enhance the progression of disease [6]. At present, there is no available treatment for *E. coli* O157:H7 infection.

The pathogenic potential of *E. coli* O157:H7 is due in large part to its ability to produce a virulence factor called Shiga toxin (Stx). Stx is a highly potent exotoxin that enters host cells and inhibits protein synthesis, resulting in cell death [7]. There are two antigenically distinct forms of Stx (Stx1 and Stx2) which share 57% sequence identity and a highly conserved overall structure [8]. Strains of *E. coli* O157:H7 can produce Stx1, Stx2, or both [9]. Stx2 is far more potent than Stx1 *in vivo* [10], [11], and the majority of fatal disease cases in humans results from Stx2-producing *E. coli* strains [12]–[15]. Stx is a member of the AB_5_ family of bacterial toxins [16], composed of an enzymatically active A-subunit that mediates damage to the host through the inhibition of protein synthesis [17], and a ring of identical B-subunits that surround the C-terminus of the A-subunit [18], [19]. The B-pentamer binds to the glycolipid globotriaosylceramide (Gb3) on the surface of host cells and delivers the A-subunit into the cell [17], [20]. Although the basis for the difference in potency between Stx1 and Stx2 is unclear, the B-subunit plays an influential role in mediating toxicity [21]–[25], suggesting that differences in B-subunit activities, such as receptor recognition or toxin internalization, are responsible for the variation in potency. Recently, using mass spectrometry techniques, Kitova et al. [26], [27] reported that Stx1B was primarily pentameric at concentrations from 5 to 85 µM. These data were supported by circular dichroism (CD) and dynamic scanning calorimetry (DSC) studies of Stx1B showing a highly thermostable pentamer [28]. In contrast, Stx2B was observed to undergo a disassembly transition in mass spectrometry studies, in which dimer, trimer, tetramer, and pentamer species were present at 65 µM, with decreasing higher-order species at lower concentrations [26], [27]. Given reports that the higher toxicity of Stx2 is due to the contributions of its B-subunit [21]–[25], further studies of Stx2B assembly and behavior are critical for advancing the understanding of Stx-mediated disease. We have completed a comparative study of the solution-state assembly and stability of Stx1B and Stx2B and point mutants using circular dichroism and analytical ultracentrifugation (AUC), complemented by the X-ray crystal structure of the Stx2B-Q40L mutant. Overall similarities were observed in the structure of the proteins, but significant differences occurred in their solution behavior. Point mutation of a key residue in the inter-subunit interface to the analogous residue found in the reciprocal isoform reversed the stability phenotype of the B-subunit, a valuable first step toward studying the role of B-pentamer instability in the elevated toxicity of Stx2.

## Materials and Methods

### Expression Constructs

pET21b(+) expression plasmids (Novagen, Darmstadt, Germany) encoding the Stx1 and Stx2 B-subunits were generated via restriction/ligation of genes amplified by polymerase chain reaction (Table 1). Plasmids were sequenced to verify proper insertion. Site-directed mutagenesis to generate Stx1B-L41Q and Stx2B-Q40L mutants was carried out using the Stratagene QuikChange® II Site-Directed mutagenesis kit (Agilent Life Sciences, Santa Clara, CA) according to the manufacturer's protocol.

**Table 1:** Strains and plasmids used in this study.

	Relevant Characteristics^a^	Reference
*E. coli* strains		
BL21(DE3)pLysS	T7 strain lysogenized with λ-DE3; pLysS plasmid; Chlor^r^	Novagen
Plasmids		
pET21b(+)	Expression vector, derived from pBR322 plasmid, Amp^r^	Novagen
pMFUC-20	pET21b(+)-Stx1B WT, Amp^r^	This study
pMFUC-21	pET21b(+)-Stx2B WT, Amp^r^	This study
pSHUC-5	pET21b(+)-Stx1B-L41Q, Amp^r^	This study
pSHUC-6	pET21b(+)-Stx2B-Q40L, Amp^r^	This study

a Amp^r^, ampicillin-resistant; Chlor^r^, chloramphenicol resistant.

### Protein Expression

pET21b(+) expression plasmids (Novagen, Darmstadt, Germany) encoding the Stx1 and Stx2 B-subunits were transformed into *E. coli* BL21(DE3)pLysS (Novagen). Transformants were cultured in Luria-Bertani (LB) broth containing ampicillin (250 µg ml^−1^) and chloramphenicol (34 µg ml^−1^) at 37°C, by shaking at 250 rpm until an OD_600_ value of 1 was reached. Cultures were cooled to 8°C and B-subunit expression was induced with 0.1 mM IPTG and 2% ethanol, followed by shaking incubation for 16 h at 20°C. Proteins were extracted by freeze-thaw and sonication.

### Protein purification

Stx1B, Stx2B, and Stx2B-Q40L were purified from induced culture lysates by ammonium sulfate precipitation (40–70%), Q Sepharose™ Fast Flow ion exchange chromatography (GE Healthcare, Uppsala, Sweden), Superdex™ 75 HiLoad 26/60 size exclusion chromatography (GE Healthcare) and Uno™ Q6R ion exchange chromatography (Bio-Rad, Hercules, CA). Stx1B-L41Q was purified from induced culture lysates by ammonium sulfate precipitation and pigeon egg white (PEW) affinity chromatography as described [29], [30]. Purity of the Stx B-subunits was verified by the presence of a single band at 8 kDa on Coomassie-stained SDS-PAGE gels. Protein concentrations were determined using the BCA Protein Assay (Pierce, Rockford, IL).

### Analytical ultracentrifugation

AUC experiments were performed in a Beckman XL-I analytical ultracentrifuge at 20°C with absorbance optics, using a four- or eight-hole rotor. All experiments were performed in 10 mM phosphate buffer with 150 mM NaCl at pH 7.4, except where otherwise noted, at wavelengths ranging from 203 nm to 280 nm. Protein loading concentrations were determined using the molar extinction coefficient calculated by ProtParam [31]. Sedimentation velocity experiments were performed at 48,000 rpm in two-channel carbon-filled epon centerpieces and data were analyzed using Sedfit [32]. Sedimentation equilibrium was performed at speeds ranging from 21,000 to 48,000 rpm and data were analyzed globally using WinNonlin (www.rasmb.bbri.org). The equilibrium association constants (K_5_, units of M^−4^) for a monomer-pentamer association were determined with the reduced buoyant molecular weight (σ) fixed at the calculated monomer value (0.4152 for Stx1B and 0.4376 for Stx2B at 21,000 rpm). The EC_50_ (monomer concentration giving 50% assembly) values reported are the fourth root of the dissociation constant, in molar units.

### Circular Dichroism

Far-UV CD experiments were carried out in an Aviv 215 circular dichroism spectrophotometer using 0.5 mm quartz cuvettes. Wavelength scans were taken from 195 to 260 nm on Stx2B wild-type (65 µM), Stx2-Q40L (65 µM), Stx1B (64 µM), and Stx1-L41Q (49 µM) in 10 mM phosphate buffer with 150 mM NaF at pH 7.4, and converted to mean residue ellipticity as described [33]. Spectra were deconvoluted using the K2D module of Dichroweb [34], [35]. Temperature melt experiments were conducted at the same concentrations and buffer conditions, except for Stx2B-Q40L, which was analyzed at 55 µM protein concentration in 10 mM phosphate buffer, 150 mM NaCl. The ellipticity at 219 nm was recorded while the temperature was increased from 25°C to 95°C in 2 degree increments with 2 minute equilibration to temperature, 5 second averaging time, and 0.2 degree temperature dead band. Fitting for T_m_ and ΔH was done in SigmaPlot 10 (Systat Software, Inc.) using a two-state model with pre- and post-transition linear changes in ellipticity [36] as a function of temperature with the following equation:
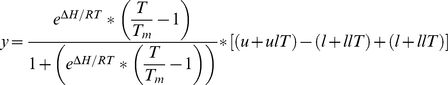
where R is the gas constant (1.987 cal·K^−1^·mol^−1^), ΔH is the enthalpy, T is the temperature in kelvin, T_m_ is the midpoint of the melting transition, u and l are the folded and unfolded limits of the ellipticity, and ul and ll are the slopes of the linear baseline changes before and after the unfolding transition, respectively [36].

### Crystallization and Structure Determination

Stx2B-Q40L was crystallized by hanging drop vapor diffusion in 100 mM HEPES pH 7.1, 200 mM magnesium chloride, and 25% PEG 3350. Crystals were grown to approximately 200 µm×200 µm×100 µm, harvested, and cryoprotected in 25% xylitol. Data was collected at APS beamline 22-ID and indexed and processed with HKL2000 [37]. Crystals nominally diffract to 2.5 Å and belong to the space group C2 with unit cell dimensions: (*a* = 121.91 Å, *b* = 69.21 Å, *c* = 103.01 Å). The structure was determined by molecular replacement using Phaser [38] with the B-subunit of Stx2 as the search model (PDB ID: 1R4P) [19]. Two Stx2B pentamers are contained within the asymmetric unit. The model was refined with refmac [39], using TLS and applying non-crystallographic symmetry restraints, and models were manually rebuilt with Coot [40]. Crystal data and refinement statistics are shown in Table 2. The structure was validated with Procheck [41] and Molprobity [42]. 95.7%, 4%, and 0.3% of the residues are in the favored, allowed, and outlier regions of the Ramachandran plot, respectively. PyMOL™ v1.2 Software (DeLano Scientific, Palo Alto, CA) was used for the creation of protein crystal structure figures (www.pymol.org).

**Table 2:** Crystal and refinement statistics.

Data Collection	Stx2B-Q40L
Beamline	APS 22-ID
Wavelength (Å)	1.000
Space Group	C2
Unit Cell (Å)	*a* = 121.91, *b* = 69.21, *c* = 103.01, *β* = 124.36°
Resolution (Å) a	30.0-2.5 (2.59-2.50)
Observed reflections	82,653
Unique reflections	24,343
Completeness (%)	98.3 (91.1)
Redundancy	3.4 (3.1)
I/σ(I)	12.18 (3.77)
R_sym_ (%) b	7.1 (23.5)

Refinement	
R_work_ (%) c	20.2%
R_free_ (%) d	26.6%
Number of atoms, protein	5,480
Number of atoms, solvent	69
RMSD bonds (Å) e	0.016
RMSD angles (°) e	1.637
B average, protein (Å^2^)	27.065
B RMSD bonds, main chain (Å^2^)	0.057
B RMSD bonds, side chain (Å^2^)	1.745

Ramachandran Plot [42]:	
Most favored	95.7%
Additional allowed	4.0%
Outlier	0.3%

a Numbers in parentheses represent the highest resolution shell.

b R_sym_ = Σ*_h_*Σ*_i_*|I*_i_*(h)-<I(h)>|/Σ*_h_*Σ*_i_*I(h).

c R_work_ = Σ||F_obs_|-|F_calc_||/|F_obs_|.

d R_free_ was calculated using a random 5% of the reflection data that were withheld from refinement.

e RMSD bonds/RMSD angles are measures of the deviation from ideal values.

## Results

**Figure 1:**
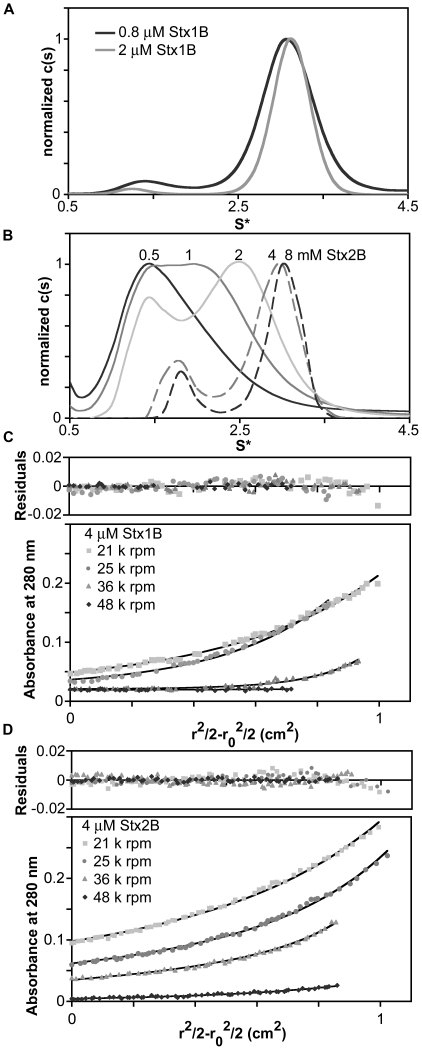
Differential solution stability of Stx1B and Stx2B pentamers. A. Sedimentation coefficient distribution plots from analytical ultracentrifugation sedimentation velocity experiments show a major peak at ∼3 S, which Sedfit estimates to have a molecular weight consistent with pentamer, at 0.8 to 2 µM loading concentrations. B. The sedimentation coefficient distributions of Stx2B at concentrations ranging from 0.5 to 8 µM demonstrate significant differences in solution state assembly. Stx2B appears to be stably pentameric at 4 to 8 µM, decreasing to mostly monomeric at 0.5 µM. C and D. Representative plots of sedimentation equilibrium data from global fits of three concentrations and four speeds in WinNonlin confirm a monomer-pentamer equilibrium for Stx1B and Stx2B. The K_5_ and EC_50_ values determined from these fits are listed in Table 3.

Reported results from mass spectrometry (MS) studies have indicated that Stx1B and Stx2B-pentamer assemblies show differential stability. Using nanoflow electrospray and Fourier-transform ion cyclotron resonance MS, Kitova et al. observed a range of assembly states from monomeric to pentameric for Stx2B at 50 to 65 µM, whereas Stx1B was observed as pentamer at all concentrations tested (from 5 to 85 µM) [26]. In order to verify the behavior of Stx1 and Stx2 B-subunits under defined solution conditions and measure accurate equilibrium association constants, AUC sedimentation velocity and sedimentation equilibrium experiments were carried out. At concentrations ranging from 0.8 µM (the detection limit of the absorbance optical system) to 2 µM Stx1B was observed to sediment with a predominant (≥85% of signal) species consistent with a pentamer (∼35+/−2.4 kDa MW) by sedimentation velocity (Figure 1A). In contrast, the extent of Stx2B pentamer assembly was reduced over that same concentration range, with varying sedimentation behavior depending on the loading concentration; pentamer assemblies were predominant at 8 and 4 µM, and disassembly occurred at concentrations of 2 µM and lower (Figure 1B).

Quantitative measurements of the Stx B-pentamer stability in solution were carried out through sedimentation equilibrium AUC experiments. Equilibrium data from four speeds and three loading concentrations were globally fitted to determine the stoichiometry and equilibrium association constants. The data were best described by a direct monomer-pentamer assembly; the inclusion of intermediate species did not improve the fits. Molar equilibrium association constants (K_5_) are listed in Table 3. The molar concentration of monomer required to achieve 50% assembly (EC_50_), was determined for Stx1B and Stx2B by taking the inverse fourth root of the molar K_5_ for each isoform. Figure 1 C and D show representative plots of data from the global fits. The EC_50_ of Stx1B is 0.043 µM, compared to 2.29 µM for Stx2B (Table 3). This reveals an approximately 50-fold higher stability of the pentameric assembly for Stx1B compared to Stx2B in the solution state.

**Table 3:** K_5_ and EC_50_ values for Stx1B, Stx2B, Stx1B-L41Q, and Stx2B-Q40L.

Isoform	K_5_ (M^−4^)	95% confidence interval	EC_50_ (µM)*	95% confidence interval
Stx1B	2.99×10^29^	1.60×10^28^ to 1.10×10^31^	0.043	0.017 to 0.089
Stx1B-L41Q	4.33×10^24^	1.33×10^24^ to 1.55×10^25^	0.693	0.504 to 0.931
Stx2B	3.64×10^22^	1.33×10^22^ to 1.06×10^23^	2.29	1.75 to 2.94
Stx2B-Q40L	7.93×10^27^	1.22×10^27^ to 6.38×10^28^	0.106	0.063 to 0.169

*The EC_50_ is the fourth root of the dissociation constant.

**Figure 2:**
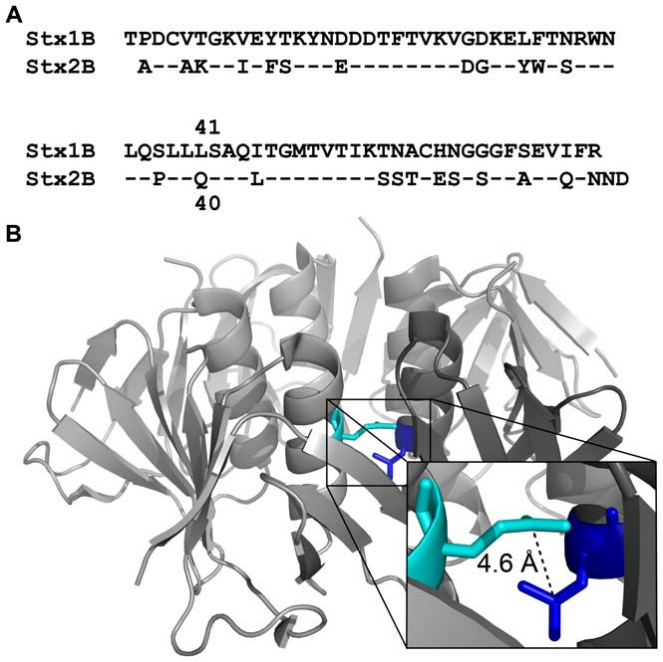
Prediction of Gln40 as a destabilizing amino acid in Stx2B. A. Alignment of Stx1B and Stx2B sequences, with identical residues identified with a dash. Residues L41 and Q40 are marked. B. Structure of the Stx2 B-pentamer (PDB ID: 1R4P) [19] indicating Gln40 (cyan) and Leu38 (blue). The polar side chain of Gln40 occupies an otherwise hydrophobic pocket at the interface between α-helices of adjacent B-subunits. Inset is a zoomed view of Leu38 and Gln40 from adjacent monomers in Stx2B. The close proximity of Leu38 and Gln40 (<5 Å) was predicted to disrupt monomer packing. In contrast, a leucine occupies position 41 in Stx1B, which may help stabilize the hydrophobic interface between B-subunits.

The fifty-fold difference in pentamer stability between Stx isoforms 1 and 2 was surprising, given that the B-subunits share 66% sequence identity. Examination of the structures for potentially destabilizing amino acids in the inter-B-subunit interface revealed a notable difference at position 40 of Stx2B, which corresponds to amino acid 41 in Stx1B (Figure 2A). Gln40 in Stx2B lies in an otherwise hydrophobic pocket (Figure 2B) and closely approaches Leu38 (Figure 2B, inset). The corresponding residue in Stx1B is Leu 41, a hydrophobic residue that may stabilize the inter-subunit interface (Figure 2A). To test the role of L41/Q40 in B-pentamer stability, we generated the reciprocal point mutants Stx1B-L41Q and Stx2B-Q40L.

**Figure 3:**
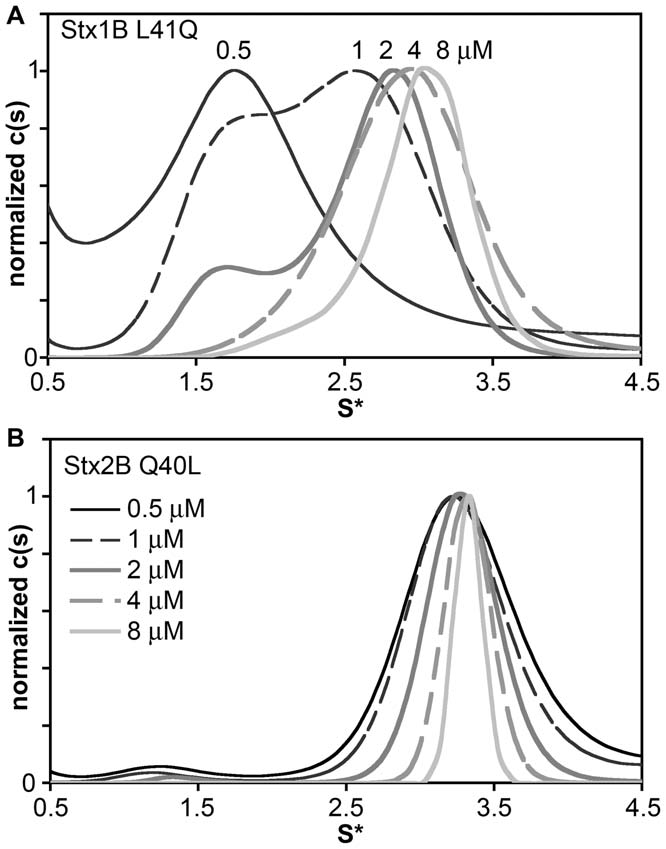
Sedimentation velocity of reciprocal point mutants. Sedimentation velocity c(s) traces for Stx1B-L41Q (A) and Stx2B-Q40L (B) demonstrate a reversal in the pentamer stability in the reciprocal mutants compared to wild-type. Stx1B-L41Q disassembles over the same concentration range that Stx1B wt retains stable pentamer assembly, and Stx2B-Q40L is stabilized relative to wt Stx2B.

**Figure 4:**
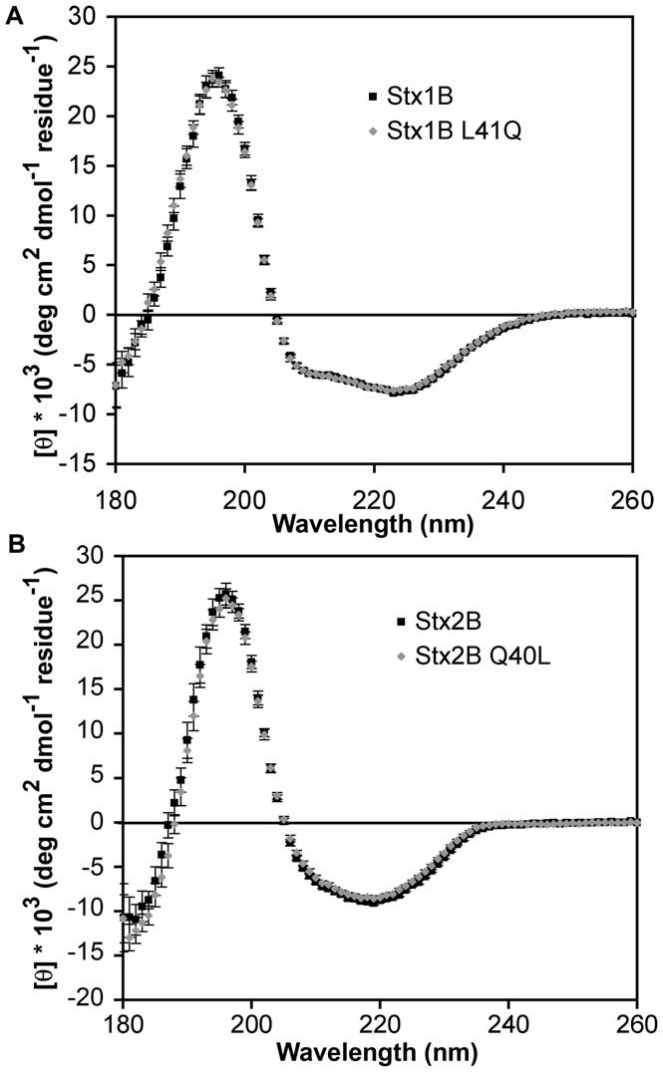
Circular dichroism spectra of wild-type and mutant proteins. A. Stx1B wild-type and L41Q mutant CD wavelength spectra. The far-UV spectra overlay, suggesting that no large-scale changes in secondary structure occur in the L41Q point mutant. B. Stx2B wild-type and Q40L mutant CD spectra. The spectra overlay, supporting the structural similarity between wild-type and Q40L mutant proteins.

AUC sedimentation velocity experiments were carried out to determine the role of residue 40/41 in the assembly of Stx B-pentamers. The Stx1B-L41Q (Figure 3A) pentamer was destabilized compared to wild-type Stx1B, disassembling to monomer or lower-order oligomers at concentrations below 4 µM. In contrast, the reciprocal mutant Stx2B-Q40L (Figure 3B) stabilized the inter-B-subunit interaction to the extent that the pentameric species predominated in sedimentation velocity experiments down to 0.5 µM, the detection limit of the absorbance optics. CD wavelength scans were used to compare the secondary structure content of wild-type and mutant B-pentamers to rule out large-scale structural deviations. The far-UV CD spectra of Stx1B and Stx1B-L41Q superimposed (Figure 4A), as did the spectra of Stx2B and Stx2B-Q40L (Figure 4B). Deconvolution of the secondary structures using the K2D module in Dichroweb [34], [35] showed no significant changes in secondary structure content. These studies indicated that any differences observed in the degree of pentamer assembly were not due to gross deviations in secondary structure caused by the mutations.

**Figure 5:**
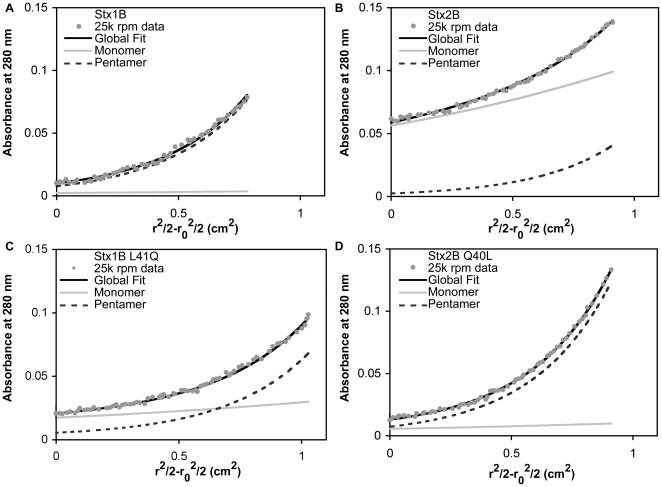
Representative species concentration plots of monomeric and pentameric B-subunits from sedimentation equilibrium data. Stx1B (A) and Stx2B-Q40L (D) show predominantly pentamer species. Stx2B (B) is primarily monomeric, whereas Stx1B-L41Q (C) retains a mixture of monomer and pentamer species. K_5_ and EC_50_ values for these data are listed in Table 3.

Sedimentation equilibrium studies quantitatively demonstrated the reversal in B-pentamer stability. Representative data curves taken from a global analysis of multiple datasets fitted to a monomer-pentamer assembly model showed the differences in assembly clearly (Figure 5). At a 2 µM loading concentration and 25K rpm rotor speed, pentamers were the major species at equilibrium for both wild-type Stx1B (Figure 5A) and Stx2B-Q40L (Figure 5D). EC_50_ values, calculated from the K_5_ values, were 0.043 and 0.106 µM for Stx1B and Stx2B-Q40L, respectively (Table 3). In contrast, Stx2B wild-type and Stx1B-L41Q showed a mix of monomer and pentamer contributions, with EC_50_ values of 2.29 µM (Stx2B) and 0.693 µM (Stx1B-L41Q). These data showed the remarkable ability of a single amino acid within the pentamer subunit interface to alter the stability of the pentameric assembly state.

**Figure 6:**
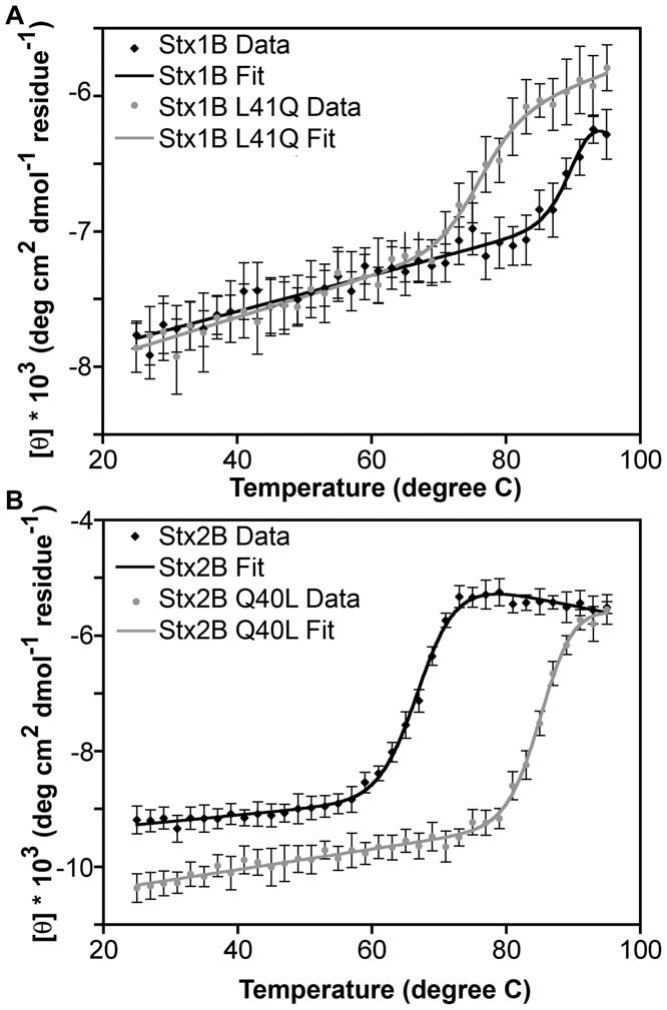
Thermal denaturation of Stx isoforms and mutants. A. Stx1B (black) and Stx1B-L41Q (gray) thermal denaturation curves. Stx1B-L41Q is destabilized (decreased melting temperature) compared to wild-type Stx1B. B. Stx2B (black) and Stx2B-Q40L (gray) thermal denaturation curves show that Stx2B-Q40L has an increased thermal stability relative to wild-type Stx2B. Parameters from the fits can be found in Table 4.

Differential pentamer stability between the Stx1 and Stx2 isoforms and mutants would be expected to alter their apparent unfolding transition, since stabilizing a subunit interface will increase the thermal stability of the oligomer. To test this, we carried out thermal denaturation studies using far-UV CD spectroscopy in order to determine the T_m_, the temperature at which 50% of the protein is denatured [36]. CD measurements of the ellipticity at 219 nm as a function of temperature revealed that the Q40L mutation raised the T_m_ of Stx2B by 18°C relative to wild-type Stx2B, whereas the reciprocal mutation L41Q in Stx1B lowered the T_m_ by at least 15°C relative to wild-type Stx1B (Figure 6) (the unfolding transition for Stx1B was not completed by 95°C and thus could not be determined precisely). Both Stx2B and Stx2B-Q40L (Figure 6B) were reversible under the scan conditions tested; however, Stx1B and Stx1B-L41Q (Figure 6A) were not, and therefore the fitted parameter values may only be used for internal comparisons. These data corroborate the AUC data, demonstrating stability reversal for the Stx1B-L41Q and Stx2B-Q40L mutants. The T_m_ and ΔH values from fits of the CD t-melt data support a two state (folded pentamer to unfolded monomer) model (Table 4), which assumes that dissociation of the pentamer is concomitant with denaturation of the monomer.

**Table 4:** Thermodynamic parameters from CD T-melt fits.

Isoform	ΔH (kcal/mol)	T_m_ (°C)
Stx1B*	−130+/−53	91.1+/−4.3†
Stx1B-L41Q*	−76.1+/−18	76.1+/−1.4
Stx2B	−79.4+/−5.2	67.2+/−0.2
Stx2B-Q40L	−100+/−9.4	85.6+/−0.7

*These melts were irreversible under the scan conditions tested.

† Protein did not completely melt under the scan conditions reported. The T_m_ is likely ≥91°C.

**Figure 7:**
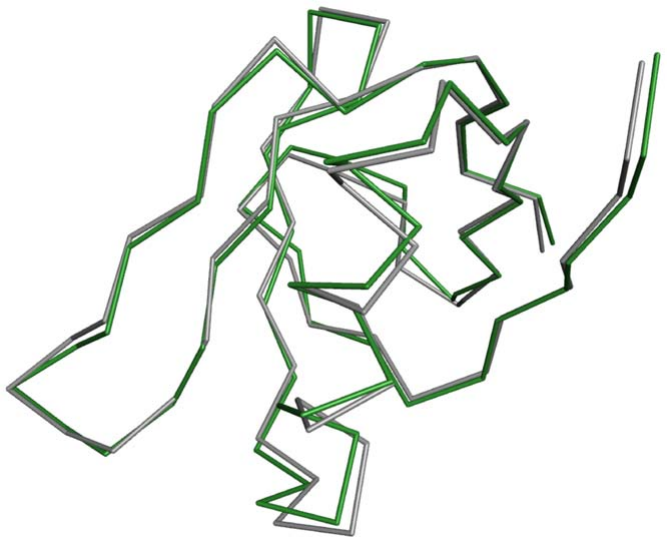
Superimposed crystal structures of Stx2B and Stx2B-Q40L subunits. Alpha-carbon traces from a representative B-subunit from the published structure of wild-type Stx2 (gray) (PDB ID: 1R4P) [19] and Stx2B-Q40L (green) overlay. The backbone RMSD of 0.260 to 0.555 Å indicates no major structural rearrangements occurred in the Stx2B-Q40L mutant.

**Figure 8:**
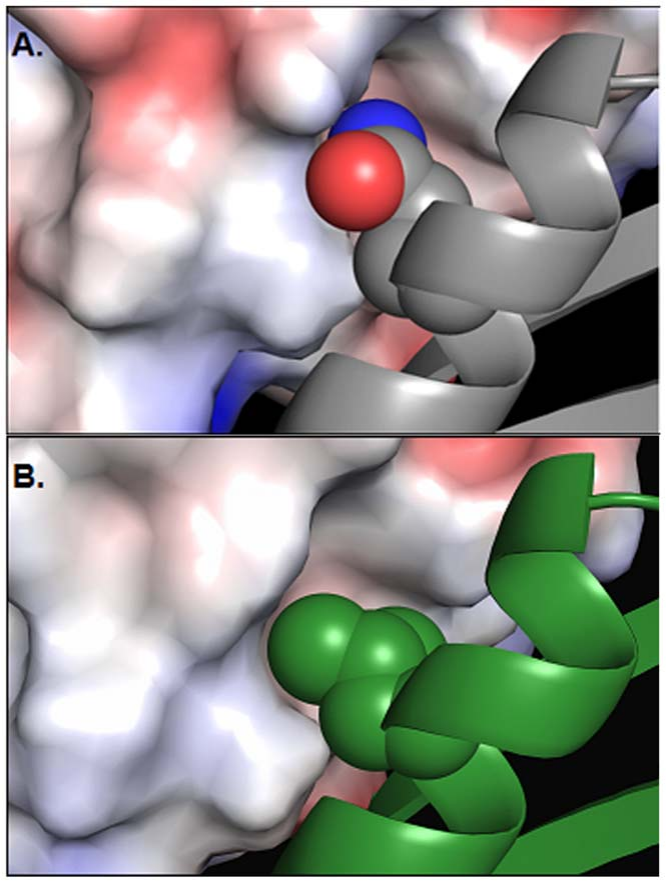
Side-chain packing for Stx2B and Stx2B-Q40L. A. A cartoon model of a single wild-type Stx2B subunit with a space-filling model of the Q40 side chain is shown in gray, and the surface of the adjacent B-subunit is colored by charge (blue  =  positive, red  =  negative, white  =  neutral). The oxygen and nitrogen atoms (colored red and blue, respectively) of the Q40 side chain lie in an unfavorable position in the inter-subunit interface. B. By contrast, the Stx2B-Q40L mutant (in green) shows the L40 side chain tightly packed into the hydrophobic pocket.

X-ray crystallographic studies (Table 2) of Stx2B-Q40L were undertaken to gain better insight into molecular packing at the inter-B-subunit interface. As expected based on the CD data, no global structural changes were observed in an overlay of the Cα traces of a single B-subunit of wild-type Stx2B and Stx2B-Q40L (average RMSD  = 0.37 Å) (Figure 7). However, a deviation in the position of the L40 side chain was observed in the mutant protein. The leucine in the mutant packs well into the hydrophobic pocket of the adjacent subunit, whereas the wild-type glutamine residue appears to be offset from the groove (Figure 8A and B). This confirmed the CD data indicating that the change in solution behavior was not due to any large-scale structural changes, but rather the destabilizing effect of the unfavorable presence of the polar side-chain of Q40 in an otherwise hydrophobic pocket.

## Discussion

Previous work has made it clear that the B-subunits are critical determinants of the differential toxicity of Stx1 and Stx2 *in vivo*. In cell-free *in vitro* translation inhibition assays, Stx1 and Stx2 A-subunit toxicities are indistinguishable, suggesting that the enzymatic activities of these subunits are not responsible for the large *in vivo* differences between the two toxins [21]–[25]. In contrast, animal models comparing wild-type and chimera Stx toxicity demonstrate that the presence of the Stx2 B-subunit is a critical determinant of lethality *in vitro* and *in vivo* [21]–[25]. Therefore, analyses of variation in the stability and assembly of Stx1B and Stx2B are fundamental to understanding the molecular basis of differential toxicity between Stx1 and Stx2. This research presents a quantitative assessment of solution-state differences in Stx B-pentamer assembly. The difference previously observed by mass spectrometry methods [26], [27] was confirmed and quantified in a direct solution-state technique. The Stx2 B-pentamer is approximately 50 times less stable in solution at pH 7.4 than Stx1 B-pentamer. The equilibrium sedimentation data could be described sufficiently by a monomer-pentamer association model without inclusion of intermediate species. Although the sedimentation coefficient distribution plots showed peaks at intermediate positions between the monomer and pentamer peaks (Figs. 1B, 3A), these do not necessarily represent true intermediate oligomeric states but rather weighted averages of the populations of monomers and pentamers in fast equilibrium relative to the time scale of the sedimentation experiment [43], [44].

Interestingly, the AUC sedimentation equilibrium K_5_ values for Stx1B and Stx2B-Q40L were within error of one another, while those of Stx2B and Stx1B-L41Q were not. This suggests that while Q40/L41 has a significant affect on B-pentamer stability, other residues are likely to also contribute. Our CD data further supports the involvement of additional residues. The T_m_ of Stx2B-Q40L is increased 18°C relative to Stx2B wild-type, but it is not within error of Stx1B. The L41Q mutation decreases the T_m_ of Stx1B by 15°C, but Stx1B-L41Q remains more stable than Stx2B. Additional interactions must account for the 5 to 9 degree offset in T_m_ between wild-type and reciprocal mutants of the opposing isoform. One candidate residue is R69, which participates in a salt bridge in Stx1B; Stx2B lacks such an interaction between the corresponding residues. Kitova et al. observed a loss of Stx1B stability upon mutation of R69 to glutamate, a charge reversal that disrupts the salt bridge [27].

The Q40/L41 side chains, although buried in the interface between B-subunits in the context of the B-pentamer, are found on the surface of each subunit monomer. Thus, the difference in thermal stability between wild-type and mutant B-subunits observed in CD experiments is not likely to be due to disruption of the hydrophobic core of each monomer. Rather, favorable packing of the inter-subunit interface stabilizes the pentamer assembly, which results in a higher T_m_ for Stx1B and Stx2B-Q40L. Thus, the two-state model used to fit the CD thermal denaturation data is an oversimplification, since it does not explicitly account for disassembly of the pentamer separately from denaturation of the monomer. However, it allows for an indirect analysis of pentamer stability via accurate determination of T_m_, the parameter that can be determined with highest confidence from CD thermal denaturation data [45]. Explicit *N*mer unfolding models are well developed for trimeric and tetrameric systems; however, higher-order models that explicitly describe oligomer dissociation linked to monomer unfolding are too complex to resolve using CD t-melts [36]. Previous studies of Stx1B thermostability have reported no improvement in fits with models that include folded monomer as an intermediate [28], [46], [47]. This does not preclude the presence of folded monomer, but merely indicates that folded monomer may be indistinguishable from folded pentamer species in the CD spectra.

Mutation of Stx1B residue L41 has previously been used to destabilize inter-subunit packing [46]. Stx1B-L41W decreased the T_m_ by 6°C in CD thermal melts. Transport of destabilized Stx1B into the cytoplasm was then assessed by MHC presentation assays, which showed that destabilization did not enhance entry into the cytoplasm. However, there is no data for how B-pentamer stability affects toxin efficacy. Destabilization of the B-pentamer may not affect entry, but a less stable toxin might more readily deliver the toxic A-subunit once internalized.

The physiological significance of the differences in B-pentamer stability is currently unclear. Numerous subtypes of Stx1 and Stx2 have been identified. The Stx2a-g subtypes display differences in potency and host range specificity. Some of the most polymorphic regions of Stx2 are involved in receptor recognition [48]–[50], suggesting that these changes confer a selective advantage, likely by altering host range specificity. In contrast, the destabilizing glutamine of Stx2 and the stabilizing leucine of Stx1 are highly conserved among their respective subtypes [49], [51], suggesting that there may be a selective advantage to maintain the phenotypes conferred by these amino acids. Studies to determine the degree to which the Stx2 holotoxin is also destabilized by the glutamine need to be performed. However, given the potency of Stx2 and the large amount of material needed to perform the biophysical characterizations reported in this study, such studies should be performed with detoxified material. Efforts to prepare genetically toxoided forms of the holotoxin are currently in progress.
